# Supplementary material for: Auxin-Producing Bacteria from Duckweeds Have Different Colonization Patterns and Effects on Plant Morphology
Source: Plants (Basel). 2022 Mar 8;11(6):721. doi: 10.3390/plants11060721 (PMC8950272; doi:10.3390/plants11060721)

Figure S1. Effect of *Microbacterium* RU1A on Arabidopsis root hairs. Inoculation of RU1A resulted in an increase in root hairs compared to the sterile control. Phase contrast microscopy was performed using the Olympus FSX100 microscope at 1/90 second exposure time with the 10x objective lens.

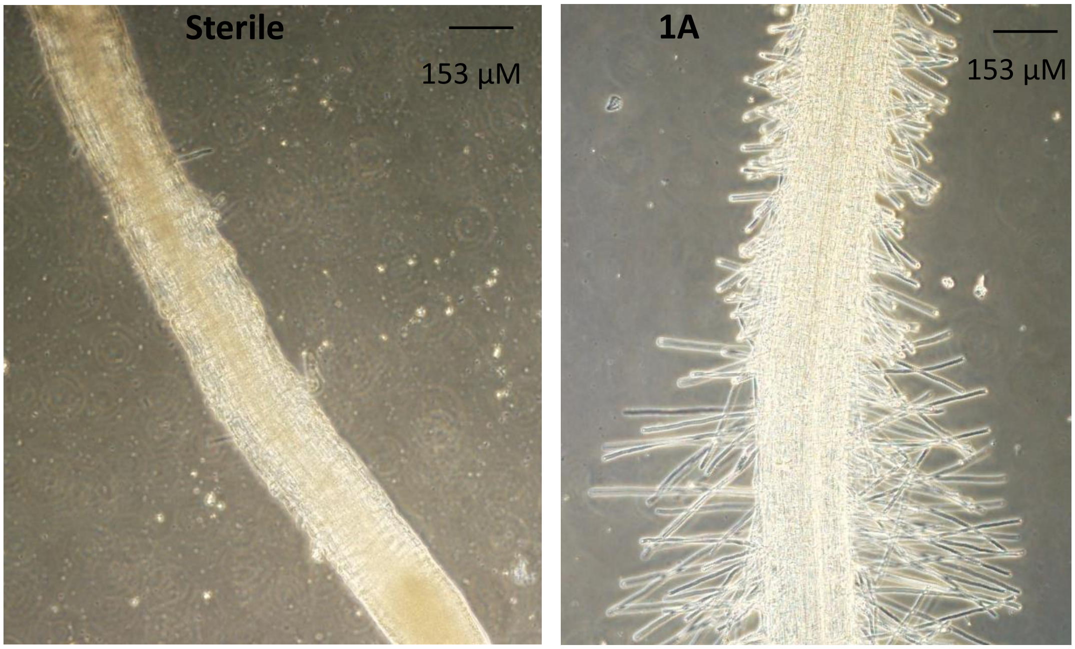

Supplement: Supplementary file 1 [file plants-11-00721-s001.zip › Figure S1.pdf]
