# Supplementary material for: Auxin-Producing Bacteria from Duckweeds Have Different Colonization Patterns and Effects on Plant Morphology
Source: Plants (Basel). 2022 Mar 8;11(6):721. doi: 10.3390/plants11060721 (PMC8950272; doi:10.3390/plants11060721)

Figure S2. IAA-producing strains differentially colonize wild type Arabidopsis leaf tissue. Maximum intensity projection of wild type leaf treated with **A)** no bacteria, **B)** *Bacillus* RU3D, **C)** *Microbacterium* RU1A, and **D)** *Microbacterium* RU33B. The microscopy channels are blue (Calcofluor White), green (Sybrgold DNA), red (chlorophyll autofluorescence) and grey (transmitted light). White arrows indicate bacteria location based on the size of the DNA-stained spots. Bacteria are shown as green spots that are smaller in size than plant nuclei.

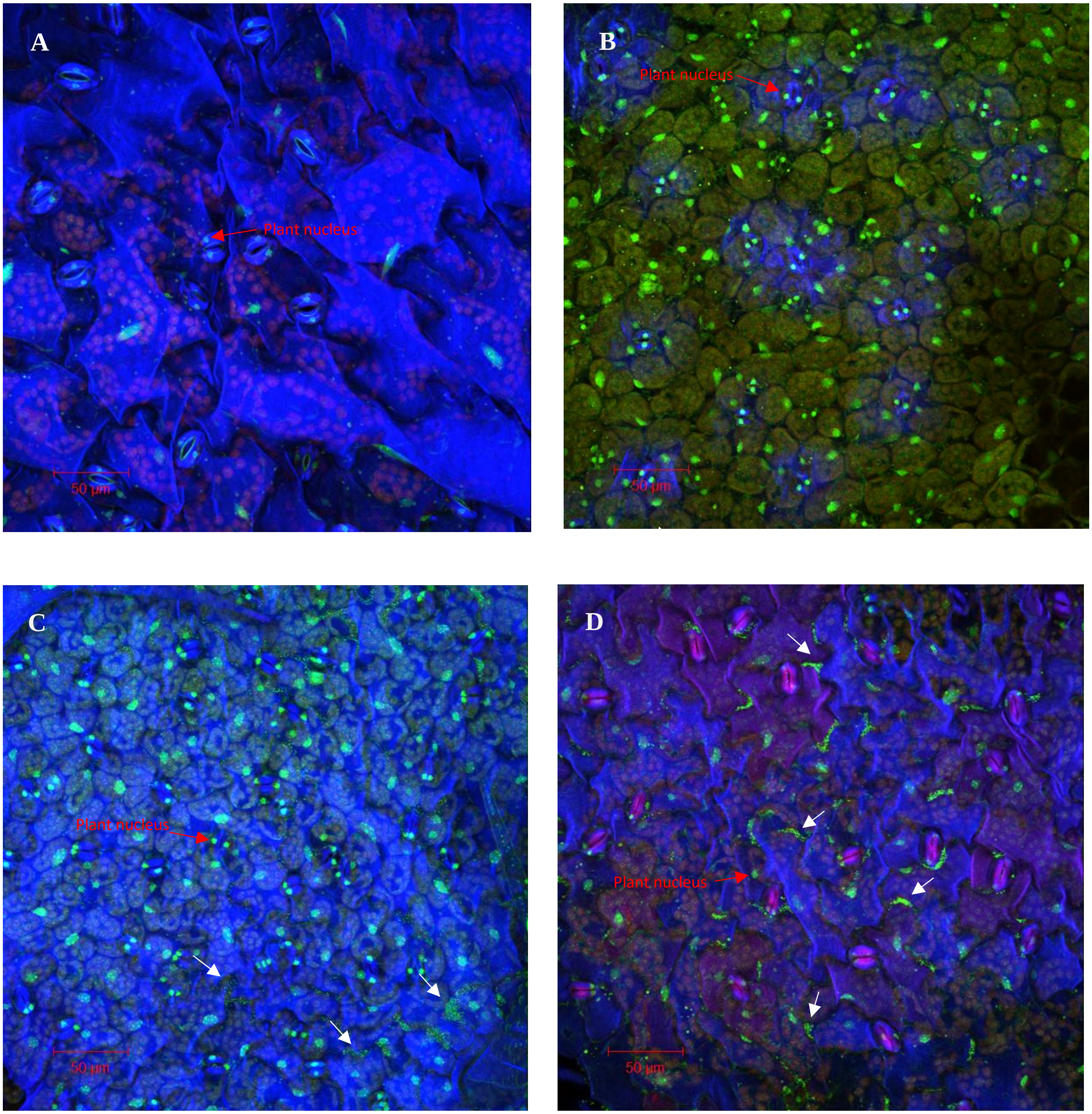

Supplement: Supplementary file 1 [file plants-11-00721-s001.zip › Figure S2.pdf]
